# A comparison of quantitative and semi-quantitative methods for assessing cartilage status and change over time; data from the osteoarthritis initiative

**DOI:** 10.1186/s12891-025-08501-6

**Published:** 2025-04-29

**Authors:** Aaron Ray, Alan D. Brett, Bright Dube, Michael A. Bowes, Emma Rowbotham, Philip G. Conaghan

**Affiliations:** 1 York and Scarborough Teaching Hospitals NHS Foundation Trust, Scarborough, UK; 2 Imorphics Ltd, Manchester, UK; 3 Leeds Institute of Rheumatic and Musculoskeletal Medicine, Chapel Allerton Hospital, University of Leeds, Chapeltown Road, Leeds, LS7 4SA UK; 4 NIHR Leeds Biomedical Research Centre, Leeds, UK; 5 Department of Radiology, Leeds Teaching Hospitals NHS Trust, Leeds, UK

**Keywords:** MOAKS, MRI, Semi-quantitative, Scoring, Quantitative, Osteoarthritis, Cartilage, Thickness, Denudation

## Abstract

**Introduction:**

The aim of this study was to compare the cross-sectional relationship and longitudinal responsiveness of the semi-quantitative MRI Osteoarthritis Knee Score (MOAKS) with automated quantitative cartilage thickness measures.

**Methods:**

Images and MOAKS scores from 297 participants with evidence of radiographic progression (groups 1 and 2) from the OAI FNIH sub-cohort were included. To facilitate direct comparison, novel quantitative measures of cartilage loss (termed Q-MOAKS) were matched to MOAKS regions. Mean normative cartilage thickness was computed for each subregion using OAI non-OA controls. The Q-MOAKS thickness loss score was based on the proportion of cartilage thickness over a subregion that was < 95% normative thickness, the denudation score was based on < 5% normative thickness. Q-MOAKS area proportions were categorised into scores as for MOAKS. Quantitative cartilage thickness (ThCtAB) was also measured in MOAKS subregions. We compared MOAKS against Q-MOAKS and ThCtAB cross-sectionally using Spearman’s rank correlation and descriptive statistics including proportions and boxplots. Longitudinally, responsiveness was assessed at 1 and 2 years using standardised response means (SRM).

**Results:**

Cross-sectionally, there was a poor correlation between MOAKS and Q-MOAKS thickness loss and denudation scores in all regions except central medial femur (cMF) and tibia (cMT) with moderate correlation for thickness loss scores: cMF, *ρ* = 0.59, (95%CI:0.51, 0.66) cMT, *ρ* = 0.58, (0.50, 0.65). In cMF, despite the broad range for the MOAKS thickness loss score = 2 (10–75% region surface area), only 56% (89/159) of knees were Q-MOAKS = 2 and 23% of MOAKS denudation = 2 were represented in Q-MOAKS = 2. In cMT, the results for similar comparisons were 61% and 66% respectively. MOAKS appeared to overestimate grades 2 and 3. Over 2-year follow-up MOAKS thickness loss and denudation scores were less responsive than Q-MOAKS in most subregions. MOAKS thickness loss was most responsive in cMT (SRM = 0.47, (95%CI:0.41, 0.54)). ThCtAB was substantially more responsive: SRM=-0.84, (-0.96, -0.73) in this region.

**Conclusion:**

Though MOAKS status scores showed reasonable correlation with quantitative measures of thickness in medial compartments, concordance between MOAKS and quantitative cartilage area loss was poor. Quantitative measures of thickness loss were substantially more responsive then MOAKS scores over a 1 and 2-year period.

**Supplementary Information:**

The online version contains supplementary material available at 10.1186/s12891-025-08501-6.

## Introduction

Magnetic Resonance Imaging (MRI) provides the best non-invasive tool for directly visualising cartilage during the progression of osteoarthritis (OA). In Disease Modifying OA Drug (DMOAD) clinical trials, OA MRI cartilage morphometry may be assessed semi-quantitatively or quantitatively. The semi-quantitative (SQ) MRI Osteoarthritis Knee Score (MOAKS) [1] assesses a range of features that are thought to be relevant to the functional integrity of the knee and the pathophysiology of OA including cartilage morphology. The MOAKS cartilage morphology score records the surface area affected by cartilage loss and denudation in 14 subregions of the knee, 12 of which form the medial and lateral tibiofemoral joint. A revision of MOAKS using ‘within grade change’, defined as structural change on imaging that is insufficient to cause a whole-grade transition [2], has been reported to improve its responsiveness [3]. In contrast to these semi-quantitative scores, quantitative cartilage measurements can be obtained using validated manual [4] or automated segmentation methods, for example by using machine learning-based technology incorporating Active Appearance Models (AAMs) [5, 6].

It has been shown that baseline cartilage damage as assessed by SQ scoring is predictive of longitudinal changes in quantitative thickness and volume measures [7, 8], and that change in cartilage damage SQ scores are associated with change in quantitative measures [9, 10, 11]. However, there is limited understanding of the detailed relationship between these semi-quantitative and quantitative methods of assessing cartilage, which measure somewhat different constructs: MOAKS assesses both the area affected by cartilage loss and the area affected by denudation, while quantitative cartilage morphology usually measures cartilage volume or thickness. The aim of this study was therefore to compare the cross-sectional relationship as well as the longitudinal responsiveness of MOAKS with quantitative cartilage thickness. To facilitate direct comparisons of the constructs assessed, we developed novel quantitative cartilage thickness loss and denudation measures designed to reflect the area of loss and area denudation ratios or scores for the tibial and femoral regions defined in MOAKS. This “Q-MOAKS” construct was designed only to compare quantitative measures to MOAKS and was not intended as a replacement of the scoring system, or as a novel clinical tool. For comparison of semi-quantitative and quantitative methods, we chose the typical time frames for OA clinical trials: 1- and 2-year follow-ups.

## Methods

### Participants

We included participants from the OA Biomarkers consortium FNIH study [12], a nested case-control study within the Osteoarthritis Initiative (OAI), a large observational study registered at ClinicalTrials.gov March 24th, 2004 (NCT00080171). Four knee outcome groups comprising 600 participants with knee Kellgren-Lawrence (KL) grade 1–3 and with combinations of radiographic and/or pain progression in an index knee have been previously defined in that study [13]. From this set of 600, we selected the 297 from the FNIH study groups 1 (194 knees) and group 2 (103 knees) that had evidence of radiographic progression, defined in the FNIH as ≥ 0.7 mm decrease in joint space width (JSW) in the medial tibiofemoral compartment from baseline to 24, 36 or 48-month follow up, with (group 1) or without (group 2) pain progression. As a non-OA control group, we used a single index knee from all subjects 46–54 years old at the OAI baseline visit that had a knee KL grade 0 with no pain (WOMAC pain subscale = 0 or 1), that were not included in the 600 participants of the FNIH Biomarkers study groups.

### MR image acquisition and quantification of measures

**Fig. 1:**
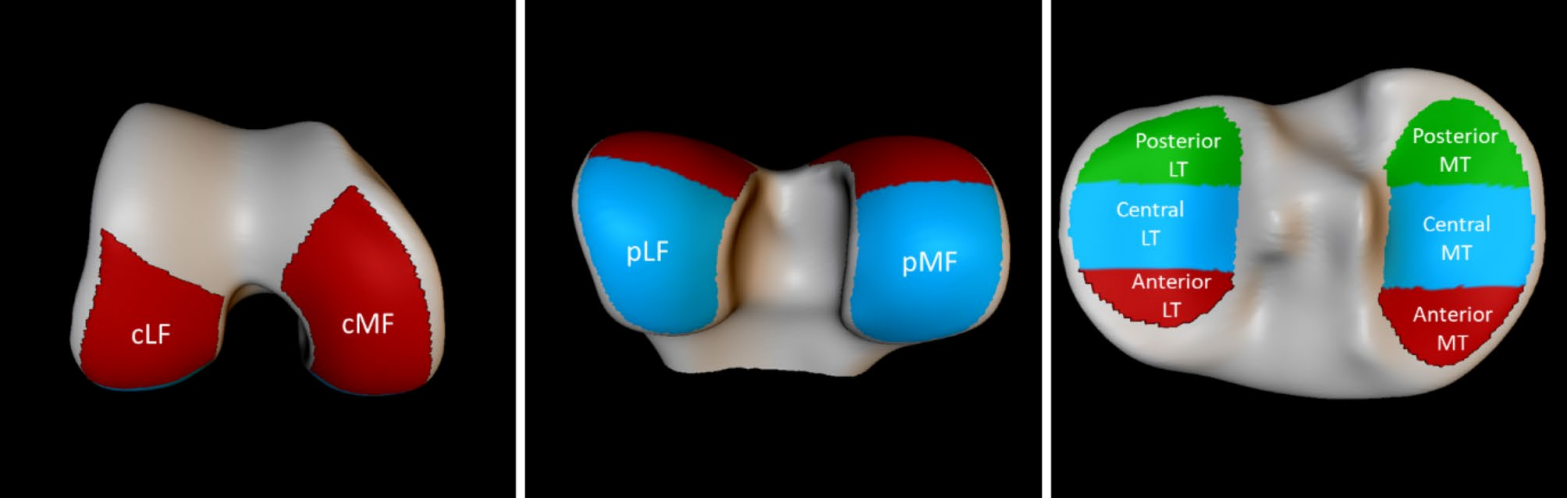
Cartilage regions defined for Q-MOAKS and ThCtAB. Cartilage regions definitions consisting of the medial and lateral central and posterior femur (cMF, cLF, pMF, pLF) subregions and the medial and lateral tibial anterior, central, posterior (aMT, cMT, pMT, aLT, cLT, pLT) subregions

For semiquantitative scoring in the FNIH study, MR images were scored unblinded to timepoint using the MOAKS scoring system centrally by two experienced musculoskeletal radiologists with 13- and 15-years’ experience of semi-quantitative assessment of knee OA. The MOAKS scores were then made publicly available through the OAI data release. MOAKS cartilage scores are composed of two components. Adapting the nomenclature of the OAI, we used the label “MOAKS Cartilage Morphology” (MCM) such that ThMCM was defined as the size of any cartilage thickness loss (partial or full thickness) as a proportion (%) of the surface area of a subregion, and dMCM was defined as the proportion (%) of full thickness cartilage loss (denudation) in this subregion. For both measures, the MOAKS cartilage scores are categorised as 0: none, 1: 0–10%, 2: 10–75% and 3: >75%. We used cartilage scores from the tibiofemoral compartments only, consisting of the medial and lateral central and posterior femur (cMF, cLF, pMF, pLF) subregions and the medial and lateral tibial anterior, central, posterior (aMT, cMT, pMT, aLT, cLT, pLT) subregions (see Fig. 1). The anterior femur was excluded because it is partly incorporated within the trochlear of the patellofemoral joint. A region is considered to have worsened if the size of the cartilage defect has changed, the amount of full thickness loss has increased, or ‘within-grade change’ has been recorded. At follow-up visits only, the OAI data scoring used a special value of 0.5 to record that although the score is the same as at the previous visit, definite worsening has occurred. This is called within-grade worsening. A special value of -0.5 is also used to record when within-grade *improvement* has occurred.

Quantitative 3D cartilage measures were derived from segmentation of MR images using Active Appearance Models (AAMS) as previously described [5, 6]. Segmentation accuracy using this method has been previously reported as mean point-to-surface distances were calculated between the manual and automated segmentations as 0.49 mm; tibia, 0.53 mm: approximately the size of one MRI voxel [14]. Test-retest reliability of cartilage thickness measurement has been reported in the cMF region with a smallest detectable difference (SDD) of 0.13 mm and a coefficient of variation (CoV) of 1.3% [6]. Validation of this method showing comparable thickness measurements to manual segmentation using image data from all 600 subjects in the OA Biomarkers consortium FNIH study in the FNIH has been previously reported [6]. The mean AAM shape was annotated to produce bone surface regions consistent with previously published MOAKS cartilage region definitions [1] so that the surfaces of the femur and tibia were divided to produce the same four femur and six tibia cartilage subregions as per MOAKS (see Fig. 1).

During auto-segmentation with AAMs, these regions are automatically propagated to each bone surface, allowing for the measurement of anatomically corresponded regions on the knee bone surfaces from each subject [5]. Average cartilage thickness (ThCtAB) in these regions was calculated by taking the mean of a set of thickness measures orthogonal to the bone surface and located at each of the dense set of AAM correspondence landmarks.

**Fig. 2:**
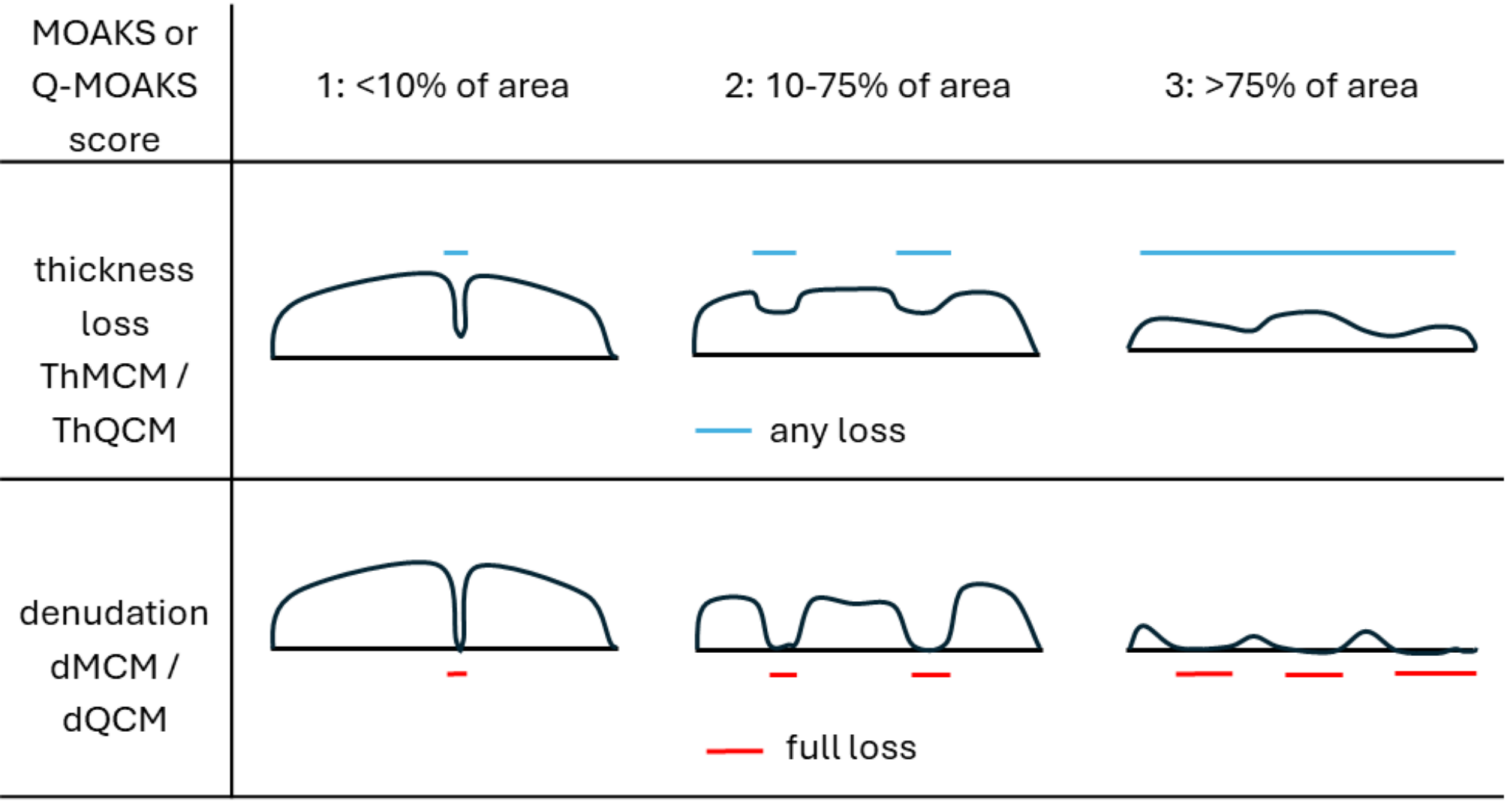
Schematic diagram of cartilage thickness loss and denudation scores with MOAKS and Q-MOAKS score nomenclature. For both measures, the MOAKS cartilage scores are categorised as 0: none, 1: 0–10%, 2: 10–75% and 3: >75%

To compare the standard SQ MOAKS scores with a quantitative equivalent value over the same cartilage region, we developed two fully-automated “Q-MOAKS” measures. We used the non-OA control group to define normative cartilage thickness values for comparison. First, normative cartilage thickness values were calculated for each surface correspondence point location as the average ThCtAB value over all the 549 OAI non-OA control subjects. We then defined cartilage loss at any location as a ThCtAB measure less than the 95th percentile of this normative ThCtAB. In the FNIH biomarker JSW progression cohort, this was then used to produce a ratio or percentage of the cartilage surface area of each MOAKS cartilage subregion that had cartilage loss (ThQCM%), and a score (ThQCM) was computed in the same way as a MOAKS score (e.g. ThQCM 2: 10–75%). We defined cartilage denudation at any location as a ThCtAB measure less than the 5th percentile of the normative ThCtAB. A ratio (percentage) full thickness loss/denudation (dQCM%) and full thickness loss/denudation score (dQCM) were then computed in a similar way to the cartilage thickness loss score, again in the FNIH JSW progression cohort. See Fig. 2 for MOAKS and Q-MOAKS nomenclature. The Q-MOAKS scores are therefore an ordinal scale, similar to MOAKS, whereas the Q-MOAKS area percentage measures are a continuous measure of the proportion of cartilage loss or denudation over one of the defined cartilage regions. These Q-MOAKS scores were used in comparison with MOAKS scores for cross-sectional analysis. For longitudinal responsiveness analysis, we used the Q-MOAKS area percentages or ratios (ThQCM% and dQCM%) as described above for comparison.

### Statistical analysis

Statistical analyses were performed using Stata version 16.0 software (StataCorp, TX, USA) and the Python pandas package version 1.3 [15]. Cross-sectionally, MOAKS and Q-MOAKS scores were compared using Spearman’s rank correlation. The association between 3D quantitative percentage area with cartilage loss and MOAKS scores in the central medial femur and tibia was explored graphically by means of boxplots. We also assessed the proportions of MOAKS 0–3 knees which achieved corresponding Q-MOAKS 0–3 categories.

Longitudinally, subregional responsiveness measured as the standardised response mean (SRM) over 1 and 2 years, was calculated to compare magnitude of change in a standardised manner, for each measure. SRMs were valid for analysis within this radiographic progression group, since expected structural changes were assumed homogenous within this group. The 95% confidence intervals (CIs) for SRMs were estimated using bootstrapping (1000 random samples with replacement).

## Results

### Cross-sectional findings

At baseline, the 297 FNIH biomarkers groups 1 and 2 included participants with a median (IQR) age of 62 (55–69) and 53% being women. The mean BMI ± SD was 30.3 ± 4.72 kg/m^2^. Our OAI baseline non-OA control group of 549 subject knees had almost the same proportion of women (52%), however they were younger and had lower BMI which might be expected in a non-OA group, see Table 1.

**Table 1:** Baseline characteristics of participants

	FNIH Groups 1 & 2 *N* = 297	OAI Non-OA Group *N* = 549
Age, years, median (IQR)	62 (55–69)	50 (48–53)
Gender, female	156 (53%)	283 (52%)
BMI, kg/m², mean (SD)	30.7 (4.7)	27.0 (4.4)
KL Grade 1	38 (13%)	
KL Grade 2	131 (44%)	
KL Grade 3	128 (43%)	
**MOAKS ThMCM (score > = 2)** ** FEMUR**		
cMF	179 (60)	
cLF	18 (6)	
pMF	110 (37)	
pLF	15 (5)	
** TIBIA**		
cMT	164 (55)	
cLT	43 (14)	
pMT	33 (11)	
pLT	83 (28)	
aMT	68 (23)	
aLT	0 (0)	
**MOAKS dMCM (score > = 2)** ** FEMUR**		
cMF	47 (16)	
cLF	1 (< 1)	
pMF	20 (7)	
pLF	0 (0)	
** TIBIA**		
cMT	29 (10)	
cLT	8 (3)	
pMT	2 (1)	
pLT	16 (5)	
aMT	8 (3)	
aLT	0 (0)	

BMI (body mass index), IQR (interquartile range), ThMCM and dMCM values are N (%)

In the central medial femur (cMF) subregion, 60% of the FNIH groups 1 and 2 participants were classed as ≥ 2 for MOAKS ThMCM and 16% for MOAKS dMCM. The distribution in other subregions is shown in Table 1. Of the 159 participants scoring 2 for MOAKS ThMCM in the central medial femur, 56% (89/159) also scored 2 for Q-MOAKS ThQCM. This was similar in the tibia (61%) (*N* = 96/157) (Table 2). In terms of denudation in the central medial femur, for those scoring 2 for MOAKS dMCM (*N* = 47), only 23% also scored 2 for q-MOAKS dQCM. In the similar subregion of the tibia, 66% of those scoring 2 on MOAKS dMCM also scored 2 for Q-MOAKS dQCM.

**Fig. 3:**
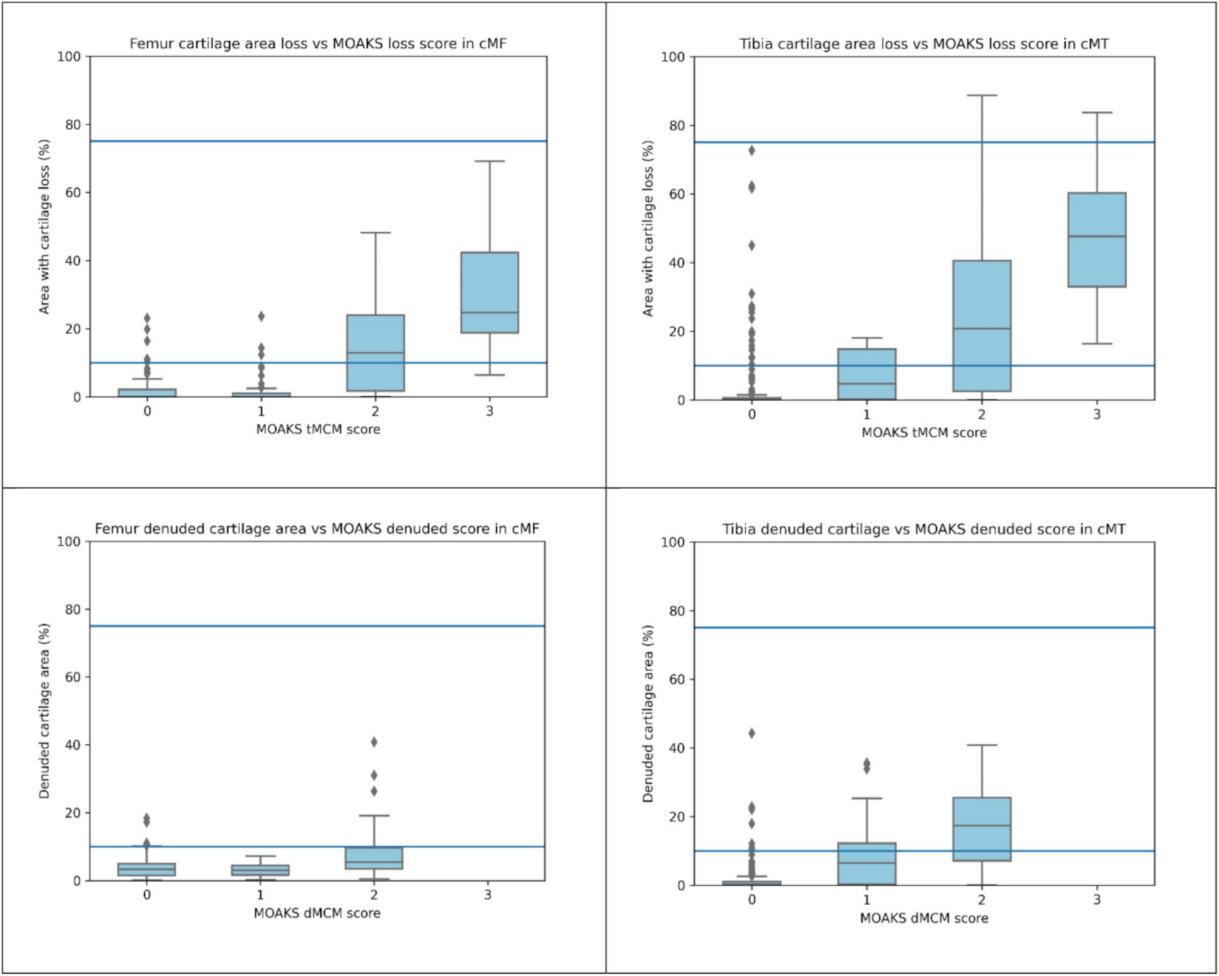
The association between 3D quantitative cartilage area percentage measures and MOAKS scores in the central medial femur and tibia. Top: comparison of cartilage thickness loss. Bottom: comparison of cartilage denudation. There were no MOAKS dMCM = 3 scores in this cohort. Horizontal blue lines indicate the MOAKS score definition thresholds (0: 0%, 1: 0–10%, 2: 10–75%, 3: >75%)

Overall, there was low or moderate correlation between MOAKS and Q-MOAKS thickness loss scores and low or negligible correlation between MOAKS and Q-MOAKS full thickness loss/denuded cartilage scores in all subregions (Table 3). The exceptions were the central medial subregions of the femur (cMF) and tibia (cMT) for the cartilage thickness loss ThMCM and ThQCM scores, where this correlation was relatively high in the femur, *ρ* = 0.59, (95%CI: 0.51, 0.66) and the tibia, *ρ* = 0.58, (95%CI: 0.50, 0.65) (Table 3). This finding was complemented by results from boxplots comparing regional quantitative percentage areas with cartilage thickness loss with MOAKS scores which showed higher median area loss values with increased MOAKS ThMCM and dMCM scores (Fig. 3). However, in general, the boxplots indicate poor agreement of MOAKS scoring in terms of the actual percentage cartilage thickness loss or denudation when compared to the MOAKS category definitions. In addition, MOAKS appeared to have a bias in that it systematically over-estimates the area of cartilage affected as measured by quantitative cartilage loss in both tibial and femoral regions.

**Table 2:** Descriptive association between MOAKS scores and Q-MOAKS scores

	Q-MOAKS ThQCM			
	0	1	2	3
**Central medial femur (cMF) MOAKS ThMCM score**				
0 (*n* = 62)	36 (58)	20 (32)	6 (10)	0 (0)
1 (*n* = 56)	35 (63)	18 (32)	3 (5)	0 (0)
2 (*n* = 159)	21 (13)	49 (31)	89 (56)	0 (0)
3 (*n* = 20)	0 (0)	1 (5)	19 (95)	0 (0)
**Central medial tibia (cMT) MOAKS ThMCM score**				
0 (*n* = 127)	92 (72)	16 (13)	19 (15)	0 (0)
1 (*n* = 6)	2 (33)	2 (33)	2 (33)	0 (0)
2 (*n* = 157)	29 (19)	25 (16)	96 (61)	7 (4)
3 (*n* = 7)	0 (0)	0 (0)	6 (86)	1 (14)

	**Q-MOAKS dQCM**			
	**0**	**1**	**2**	**3**
**Central medial femur (cMF) MOAKS dMCM score**				
0 (*n* = 215)	0 (0)	205 (95)	10 (5)	0 (0)
1 (*n* = 35)	0 (0)	35 (100)	0 (0)	0 (0)
2 (*n* = 47)	0 (0)	36 (77)	11 (23)	0 (0)
3 (*n* = 0)	0 (0)	0 (0)	0 (0)	0 (0)
**Central medial tibia (cMT) MOAKS dMCM score**				
0 (*n* = 233)	81 (35)	145 (62)	7 (3)	0 (0)
1 (*n* = 35)	6 (17)	14 (40)	15 (43)	0 (0)
2 (*n* = 29)	1 (3)	9 (31)	19 (66)	0 (0)
3 (*n* = 0)	0 (0)	0 (0)	0 (0)	0 (0)

Values are N (%)

**Table 3:** Correlation between MOAKS scores and Q-MOAKS scores in the femur and tibia

	Femur subregions			
	cMF	cLF	pMF	pLF
**ThMCM/ThQCM**	0.59 (0.51, 0.66)	0.06 (-0.06, 0.17)	0.43 (0.33, 0.52)	0.24 (0.13, 0.34)
**dMCM/dQCM**	0.19 (0.08, 0.30)	-0.02 (-0.13, 0.09)	-0.03 (-0.14, 0.09)	-0.12 (-0.23, 0.00)

	Tibia subregions					
	cMT	cLT	pMT	pLT	aMT	aLT
**ThMCM/ThQCM**	0.58 (0.50, 0.65)	0.44 (0.32, 0.54)	0.25 (0.14, 0.35)	0.18 (0.07, 0.29)	0.31 (0.20, 0.41)	n/a
**dMCM/dQCM**	0.46 (0.36, 0.54)	-0.08 (-0.19, 0.03)	0.07 (-0.05, 0.18)	-0.01 (-0.13, 0.10)	0.08 (-0.04, 0.19)	n/a

Values are Spearman’s rank correlation coefficient ρ (95% CI)

### Longitudinal findings: responsiveness

The SRMs are reported in Table 4 for the main cartilages thickness outcomes of ThMCM, ThQCM% and ThCtAB at 1 and 2-years respectively. Results of denudation scores and ratios are presented in Supplemental Table S1. In the subregional analyses, MOAKS ThMCM and dMCM were less responsive than Q-MOAKS ThQCM% and dQCM% and the quantitative thickness ThCtAB measures in most subregions. At 2-year follow-up, the greatest responsiveness for all measures was seen in the central medial femur region (cMF) with the quantitative measures being more responsive than semi-quantitative scoring; SRM = +0.73 (+0.64, +0.83) for Q-MOAKS ThQCM and SRM = -0.84 (-0.96, -0.73) for ThCtAB vs. SRM = +0.47 (95% CI: +0.41, +0.54) for MOAKS ThMCM. In the tibia at 2-year follow-up, similar findings were seen: responsiveness in the central medial tibia (cMT) was SRM = +0.49 (+0.38, +0.60) for Q-MOAKS ThQCM% and SRM = -0.73 (-0.85, -0.62) for ThCtAB compared to SRM = + 0.39 (+0.33, +0.45) for MOAKS ThMCM. Two-year responsiveness in all other subregions of the femur and tibia was lower than reported for cMF and CMT. The trends for 1-year responsiveness for all measures was similar to 2-years but of a much lower magnitude. There was no consistent pattern discernible in the comparison of SRMs related to MOAKS and Q-MOAKS thickness loss vs. denudation (Table 4).

**Table 4:** Responsiveness (SRM) of standard cartilage MOAKS scores, Q-MOAKS ratios and quantitative cartilage thickness at one-year and two-year follow-up. Note: ThCtAB SRMs are negative due to loss of cartilage whereas score and ratio changes are positive

	YEAR ONE YEAR TWO					
	ThMCM	ThQCM%	ThCtAB	ThMCM	ThQCM%	ThCtAB
				FEMUR		
	**MOAKS**	**Q-MOAKS**	**Thickness**	**MOAKS**	**Q-MOAKS**	**Thickness**
cMF	0.15 (0.02, 0.27)	0.36 (0.25, 0.47)	-0.32 (-0.44, -0.20)	0.47 (0.41, 0.54)	0.73 (0.64, 0.83)	-0.84 (-0.96, -0.73)
cLF	0.00 (-0.11, 0.12)	0.09 (-0.04, 0.18)	-0.03 (-0.14, 0.08)	0.11 (0.00, 0.18)	0.24 (0.17, 0.31)	-0.22 (-0.32, -0.11)
pMF	0.21 (0.11, 0.29)	0.18 (0.06, 0.31)	-0.07 (-0.19, 0.04)	0.46 (0.39, 0.53)	0.41 (0.33, 0.51)	-0.37 (-0.48, -0.28)
pLF	0.06 (0.00, 0.10)	0.09 (-0.03, 0.17)	0.02 (-0.09, 0.15)	0.08 (0.00, 0.12)	0.18 (0.11, 0.26)	-0.20 (-0.30, 0.08)

				**TIBIA**		
	**MOAKS**	**Q-MOAKS**	**Thickness**	**MOAKS**	**Q-MOAKS**	**Thickness**
cMT	0.16 (0.05, 0.26)	0.28 (0.18, 0.38)	-0.40 (-0.52, -0.30)	0.39 (0.33, 0.45)	0.49 (0.38, 0.60)	-0.73 (-0.85, -0.62)
cLT	0.03 (-0.07, 0.16)	0.14 (0.04, 0.24)	-0.23 (-0.34, -0.13)	0.19 (0.14, 0.25)	0.22 (0.12, 0.32)	-0.47 (-0.59, -0.36)
pMT	0.06 (-0.06, 0.16)	0.22 (0.15, 0.30)	-0.19 (-0.32, 0.08)	0.19 (0.12, 0.24)	0.38 (0.30, 0.45)	-0.48 (-0.56, -0.34)
pLT	-0.06 (-0.13, 0.06)	0.09 (-0.02, 0.20)	-0.21 (-0.31, -0.10)	-0.04 (-0.13, 0.10)	0.23 (0.12, 0.34)	-0.52 (-0.63, -0.42)
aMT	0.15 (0.07, 0.21)	0.13 (0.01, 0.24)	-0.09 (-0.21, 0.03)	0.31 (0.24, 0.38)	0.28 (0.17, 0.39)	-0.34 (-0.45, -0.23)
aLT	n/a -	-0.03 (-0.13, 0.10)	-0.06 (-0.17, 0.05)	n/a -	0.13 (0.04, 0.20)	-0.33 (-0.43, -0.23)

Values are SRM (95% CI)

## Discussion

We first explored the cross-sectional relationship of MOAKS with quantitative cartilage morphometry measures and then examined their responsiveness at 1 and 2-years. We found a moderate relationship between MOAKS thickness loss (ThMCM and Q-MOAKS ThQCM) scores in the cMF and cMT subregions only, other regions exhibited weak or no correlations. There was weak or no relationship present in any region for the denuded (dMCM and dQCM) scores. MOAKS appeared to overestimate thickness loss for MOAKS grades 2 and 3. From the OA Biomarkers consortium FNIH study, we chose a cohort (group 1 and group 2) that was selected to demonstrate radiographic progression, defined as ≥ 0.7 mm decrease in JSW in the medial tibiofemoral compartment during 4-year follow up. This cohort was therefore most likely to demonstrate cartilage loss or denudation during the 1-year and 2-year follow up periods that we analysed. Quantitative measures of thickness loss were substantially more responsive than MOAKS.

The MOAKS category 2 score (either cartilage loss or denudation) contains the widest range of values (10–75%) compared to other scores. Therefore, we expected a high proportion of knees scored as MOAKS 2 to equate to a Q-MOAKS score 2. However, for thickness loss (ThMCM), only 56% and 61% of MOAKS 2 knees also scored Q-MOAKS 2 (ThQCM) in the cMF and cMT subregions, with 23% and 66% in the cMF and cMT subregions for denudation (dMCM) compared with dQCM. In the remaining knees, the majority of MOAKS 2 scores were classified as Q-MOAKS 1, indicating MOAKS may overcall the area of cartilage loss and denudation in a high proportion of cases. The boxplots of Fig. 3 confirm this: there are MOAKS 2 thickness loss grades that correspond to actual areas of loss of < 10% in the cMF and cMT regions. In addition, the MOAKS 3 grades do not appear to accurately represent proportional areas of loss of > 75%, although these are few in number and so this result should be treated with caution. These findings suggest that an accurate semi-quantitative evaluation of cartilage status by area of loss or denudation may be difficult to achieve.

MOAKS assessment relies on qualitative radiological evaluation and although in general, diagnostic accuracy for full thickness lesions is better than earlier grade cartilage lesions [16, 17], here we observed a better relationship of the quantitatively-derived scoring with thickness loss (ThMCM) compared to denudation (dMCM). However, this may have been due to the comparison of categorisation of the *area* of loss or denudation, which may be more challenging than comparison of a decision regarding the depth of cartilage lesion in the ICRS and Outerbridge chondral classification systems in previous arthroscopic studies.

Longitudinal responsiveness was compared using SRMs. To maximise the responsiveness of MOAKS we used the within-grade scoring method. Within-grade change has been proposed as a way to increase sensitivity to change [3] and can overcome the ceiling effect in MOAKS grade 3 knees [18] compared to standard whole-grade definitions of cartilage progression. For example, a MOAKS grade 2 for size and full-thickness cartilage loss is defined as 10–75% of the surface area which reflects a wide range of severity and change within this category would not be recorded with standard definitions. The most responsive subregions for MOAKS were the cMF and cMT but these exhibited small (Cohen’s definition) responsiveness at 1-year for both thickness loss (ThMCM) and denudation (dMCM) and at 2-years for thickness loss; and moderate responsiveness for denudation at 2-years. Q-MOAKS was more responsive than MOAKS overall and achieved moderate responsiveness in the cMF region for thickness loss (ThQCM) but not for denudation (dQCM) at 2-years. In many subregions, denudation was more responsive than cartilage thickness loss for MOAKS. However, this was not the case for Q-MOAKS which showed some variability in this regard. There were a small number of instances of scores indicating cartilage increase: over 1 year MOAKS scores decreased in the pLT region and Q-MOAKS scores decrease in the aLT region. Over 2 years, the MOAKS score at the pLT region decreased. There are no instances of the quantitative cartilage thickness measure (ThCtAB) increasing. Given that the actual decreases in these scores are very small, it is likely that these values are simply reflecting errors in the scores or measures.

Quantitative cartilage thickness (ThCtAB), measured in central tibial and femoral subregions, was the most responsive measure in all regions at both 1 and 2-years and achieved large responsiveness in the cMF subregion. This is consistent with prior studies which have shown this medial region to be most responsive [19]. ThCtAB provides an efficient summary measure of both cartilage thinning and denudation [20], however here we compared its responsiveness to cartilage area loss only, which is more comparable to cartilage thinning than denuded area. We ensured that the spatial boundaries of cMF had the same definition for MOAKS, Q-MOAKS and ThCtAB, and so it is very unlikely that any differences could be attributed to differences in spatial boundaries. The fact that ThCtAB is considerably more responsive than either MOAKS or Q-MOAKS in most subregions indicates that the use of cartilage loss area change, as opposed to thickness loss change, as a measure is simply less responsive. It is possible that this is because cartilage lesions tend to be localised and progress longitudinally by becoming deeper rather than becoming wider or they tend towards becoming deeper before they become wider.

Previously, an association between changes in a SQ cartilage defect grade and changes in cartilage volume over 2 or 2.9 years have been seen in cohorts of older adults and the adult children of parents with previous joint replacement [9, 10]. Although the cartilage volume loss per annum was around 2–3%, the majority of cartilage defects remained stable during the period in the older adults cohort [10]. A more recent study that compared MOAKS cartilage scores and cartilage thickness in the OA Biomarkers consortium FNIH cohort showed that knees with any increase in SQ cartilage scores showed more quantitative cartilage loss compared to knees that remained stable [11]. However, these studies did not explore MOAKS responsiveness which the current study has done. Our findings are consistent with previous studies which have shown quantitative techniques to be more responsive or sensitive to change [21, 22] than semi-quantitative cartilage morphometry using the Whole Organ Magnetic Resonance Imaging Score (WORMS). Although WORMS has similarities to MOAKS and the subregional definitions are the same, the cartilage scoring categorisation utilises changes in cartilage thickness, full thickness lesions and changes in the MRI signal. Meta-analysis has previously reported pooled responsiveness of semi-quantitative WORMS cartilage scoring with an SRM of 0.55 (95%CI: 0.47, 0.64) in the combined medial tibiofemoral region and 0.37 (0.18, 0.57) in the combined lateral tibiofemoral region [23]. However, it should be noted that two of the three studies that this pooled result was derived from used data from the Boston OA of the Knee Study with follow-up at 30 months [24, 25], suggesting that an SRM over 24 months would be similar to what we found for MOAKS in our study for these regions.

We constructed a quantitative equivalent to MOAKS (Q-MOAKS) using the same anatomical boundaries as MOAKS to explore how scores based on semi-quantitative and quantitative cartilage measures compare. Q-MOAKS was not designed as a measurement tool itself (and we would not advocate it’s use for that purpose), but to understand the relative responsiveness and associations of semi-quantitative cartilage scores and quantitative cartilage thickness measures. With reference to Fig. 1, it may be noted that the 3 regions on each tibial plateau do not appear to be similar sized tertiles, which might be expected because according to the definition of MOAKS, the whole of the tibial plateau is split into 3 equal size tertiles. This definition was followed, however, the proportion of the plateau which is actually covered by cartilage on average is quite different in each region. The central region has more cartilage cover. Since the Q-MOAKS and MOAKS scoring systems are actually scoring the same cartilage regions, it would not be expected that this would impact the accuracy of the Q-MOAKS scores. This may, however, have implications for the way scores should be combined, perhaps using weights, for aggregated regions.

There are a several limitations of this work. The normative cartilage thickness values for the determination of thinning and denudation we derived from the non-OA group was not stratified by sex or height, both of which may alter expected thickness. We might also have stratified by age, but this was not possible as there was little or no overlap between the non-OA and FNIH biomarker groups. These stratifications might have made the Q-MOAKS scores more accurate. However, our intention was not to define a set of “normal” cartilage thickness measures, but to compute simple thresholds to determine probable thinning and denudation. Future work might improve upon this. The study selected participants chosen for the presence or absence of structural progression and may not represent a broader population sample. In addition, the absence of MOAKS grade 3 denuded knees in this sample precludes extension of our findings to severely denuded knees. We did not attempt to produce aggregated or summed scores for larger regions such as the whole medial tibiofemoral region. While it may be interesting to compare the responsiveness of combined regions, the most appropriate way to produce such aggregated scores for comparison is debatable. This study also only focusses on cartilage; OA is considered a whole joint-organ disease and therefore semi-quantitative assessment is beneficial as it provides a multi-tissue assessment. Semi-quantitative assessment may therefore continue to serve as a complimentary tool to quantitative assessment [26]. Further studies are needed to compare semi-quantitative and quantitative assessment of other OA tissues such as osteophytes and bone marrow lesions.

## Conclusions

In conclusion, there is a moderate or poor cross-sectional relationship between matched subregions of quantitative cartilage area thickness loss or denudation, and semi-quantitative cartilage scores assessed with MOAKS. The accuracy of categorising proportional areas affected by thickness loss or denudation for MOAKS scoring appears to be quite poor. However, the responsiveness of the Q-MOAKS measure developed for this analysis which will do this categorisation more accurately, is still lower than quantitative ThCtAB and more similar to MOAKS, particularly at 2 years. It would appear that the construct of area change in MOAKS scoring, as opposed to thickness change from quantitative measures, reduces the responsiveness of the semi-quantitative approach. This would be the case if cartilage lesions tend to progress by becoming deeper rather than wider, which is supported by the fact that Q-MOAKS ThMCM, despite being a quantitative measure, is less responsive than ThCtAB in most regions at 2 years. The area affected by cartilage loss is a less responsive measure of OA progression, whether assessed semi-quantitatively by experts, or by accurate quantification of cartilage. The responsiveness of quantitative cartilage thickness ThCtAB measures are substantially superior to MOAKS scores as an assessment of cartilage change. This has importance in the powering and conduct of DMOAD clinical trials with cartilage endpoints.

## Electronic Supplementary Material

Below is the link to the electronic supplementary material.

Supplementary Material 1

Supplementary Material 2

## Data Availability

The images and MOAKS datasets analyzed during current study are available in the OAI repository, https://nda.nih.gov/oai/. Q-MOAKS datasets used and/or analysed during the current study are available from the corresponding author on reasonable request.
