# Supplementary material for: A comparison of quantitative and semi-quantitative methods for assessing cartilage status and change over time; data from the osteoarthritis initiative
Source: BMC Musculoskelet Disord. 2025 Apr 29;26:426. doi: 10.1186/s12891-025-08501-6 (PMC12042560; doi:10.1186/s12891-025-08501-6)
Supplement: Supplementary file 1 — Supplementary Material 1 [file 12891_2025_8501_MOESM1_ESM.docx]

**Supplemental Data**

***Table S1. Responsiveness (SRM) of standard cartilage MOAKS scores and Q-MOAKS ratios at one-year and two-year follow-up for***

|  | **YEAR ONE** | | **YEAR TWO** | |
| --- | --- | --- | --- | --- |
|  | **dMCM dQCM%** | | **dMCM dQCM%** | |
|  | **FEMUR** | | | |
|  | **MOAKS** | **Q-MOAKS** | **MOAKS** | **Q-MOAKS** |
| cMF | 0.30  (0.22, 0.37) | 0.18  (0.09, 0.26) | 0.55  (0.48, 0.62) | 0.43  (0.36, 0.49) |
| cLF | 0.08  (-0.03, 0.16) | 0.12  (0.02, 0.20) | 0.15  (0.04, 0.22) | 0.28  (0.23, 0.36) |
| pMF | 0.16  (0.05, 0.23) | 0.10  (-0.02, 0.20) | 0.29  (0.22, 0.35) | 0.34  (0.26, 0.42) |
| pLF | 0.12  (0.06, 0.18) | 0.09  (-0.02, 0.19) | 0.14  (0.08, 0.19) | 0.27  (0.20, 0.36) |
|  | **TIBIA** | | | |
|  | **MOAKS** | **Q-MOAKS** | **MOAKS** | **Q-MOAKS** |
| cMT | 0.37  (0.31, 0.45) | 0.25  (0.14, 0.35) | 0.55  (0.49, 0.62) | 0.44  (0.37, 0.52) |
| cLT | 0.11  (0.00, 0.19) | 0.14  (0.03, 0.23) | 0.19  (0.12, 0.24) | 0.29  (0.21, 0.38) |
| pMT | -0.06  (-0.10, 0.00) | 0.19  (0.09, 0.29) | 0.10  (0.00, 0.15) | 0.42  (0.34, 0.52) |
| pLT | 0.06  (-0.05, 0.18) | 0.02  (-0.10, 0.12) | 0.13  (0.04, 0.21) | 0.30  (0.20, 0.40) |
| aMT | 0.19  (0.11, 0.26) | 0.12  (0.02, 0.22) | 0.27  (0.21, 0.33) | 0.29  (0.20, 0.38) |
| aLT | n/a  - | 0.04  (-0.09, 0.14) | n/a  - | 0.31  (0.23, 0.39) |

Values are SRM (95% CI)
