# Supplementary material for: A comparison of quantitative and semi-quantitative methods for assessing cartilage status and change over time; data from the osteoarthritis initiative
Source: BMC Musculoskelet Disord. 2025 Apr 29;26:426. doi: 10.1186/s12891-025-08501-6 (PMC12042560; doi:10.1186/s12891-025-08501-6)
Supplement: Supplementary file 2 — Supplementary Material 2 [file 12891_2025_8501_MOESM2_ESM.pdf]

A Comparison of Quantitative and Semi-quantitative Methods for Assessing Cartilage Status and Change Over Time; Data from the Osteoarthritis Initiative

Editor comments

The authors aim to elucidate our understanding of osteoarthritis and osteoarthritis progression by developing novel quantitative markers that can be extracted from MRI. There are several issues with the manuscript that are mentioned in the reviewers' comments that need to be addressed. There are significant revisions required to make this manuscript suitable for publication.

Editor Comment	Response / Action
1. In addition, it is unclear whether the selection of cases (x-ray progression vs pain progression) are a homogeneous group it would be interesting to separate these two groups to assess changes.	Our apologies if we were not clear about inclusion. The purpose of this study is to explore the relationship between semi-quantitative (SQ) and quantitative cartilage measures derived from MR images. As such, we wanted to test the quantitative and SQ methods on knees with probable cartilage loss. Therefore, we chose the two groups from the FNIH biomarkers cohort that had radiographic progression and therefore probable cartilage loss. One of these groups only had x-ray progression (N=103) and the other had x-ray and pain progression (N=194). Separating out these groups would produce a relatively small sample size in the group with only x-ray progression, which, given the slow progression of OA and minimal cartilage loss over two years, we feel is unlikely to produce easily interpretable results.<br><br>Action: We have clarified the fact that there was x-rays progression in the two groups that we chose:<br>• Lines 90-94: From this set of 600, we selected the 297 from the FNIH study groups 1 (194 knees) and group 2 (103 knees) ~~with~~ that had evidence of radiographic progression, defined in the FNIH as ≥ 0.7mm decrease in joint space ~~narrowing~~ width (JSW~~N~~) in the medial tibiofemoral compartment from baseline

	to 24, 36 or 48-month follow up, with (group 1) or without (group 2) pain progression.
2. It is unclear whether only 1 knee per patient was used in the case group. The authors clearly state that only 1 knee per patient was used for the control group.	We thank for the editor for pointing out this omission and have now clarified our inclusion criteria in the methods section.<br><br>Action: We have made the following changes in the METHODS/Participants section:<br>• Line 90: …progression in an index knee have been previously defined in that study [13].<br>• Line 95: As a non-OA control group, we used a single index knee…
3. The authors used 549 non-OA controls from the OAI study, where 95% of the threshold is used to define disease, so that 5% of the controls have disease by definition. However, If this is done recursively, then the non-OA controls will always have patients with disease.	We apologize if we were not clear. The non-OA cohort were selected based only on no symptomatic or radiographic signs of disease. We then sought to define a threshold that would provide a possible indication of thinning (mean regional cartilage thickness is <95% of normal range); and another threshold that would provide a possible indication of complete denudation (mean regional cartilage thickness is <5% of normal range). These were used to estimate thinned or denuded regions in the two FNIH biomarkers JSW progression cohorts, but not in the non-OA controls.<br><br>In terms of recursion, we presume that this a reference to the bootstrapping method we used to numerically derive 95% CIs for the SRMs in the longitudinal analysis. So, the recursion is done not on the non-OA control data cohort, but on the set of longitudinal SQ differences that have been calculated in our FNIH biomarkers JSW progression cohort.<br><br>Action: we have updated the Methods section to clarify the use of the non-OA group for normative thickness measures:<br>• Lines 141-148: To compare the standard SQ MOAKS scores with a quantitative equivalent value over the same cartilage region, we developed two “Q-MOAKS” measures. We used the non-OA control group to define normative cartilage

	thickness values for comparison. First, normative cartilage thickness values were calculated for each surface correspondence point location as the average ThCtAB value over all the 549 OAI non-OA control subjects. We then defined cartilage loss at any location as a ThCtAB measure less than the 95th percentile of this normative ThCtAB. In the FNIH biomarker JSW progression cohort, t~~T~~his was then used to produce a ratio or percentage of the cartilage surface area of each…
4. No test-retest studies of reliability of QMOAHS and ThCTAB. No reliability of segmentation presented.	We thank the editor for bringing this omission to our attention and have added data from previous studies with citations. This addresses the omission we made on the reliability of ThCtAB. However, we do not believe that test-retest reliability figures for Q-MOAKS would be appropriate since this is measure is converted from proportional cartilage areas to an ordinal score.<br><br>Action: we have added figures and citations in the METHODS section:<br>• Lines 119-126: Appearance Models (AAMS) as previously described [5], [6]. Segmentation accuracy using this method has been previously reported as mean point-to-surface distances were calculated between the manual and automated segmentations as 0.49mm; tibia, 0.53mm: approximately the size of one MRI voxel [14]. Test-retest reliability of cartilage thickness measurement has been reported in the cMF region with a smallest detectable difference (SDD) of 0.13mm and a coefficient of variation (CoV) of 1.3% [6]. The mean AAM shape…
5. The acronyms are very confusion in this manuscript. Usually acronyms follow the words they replace. For example, COrona VIrus Disease 2019 is abbreviated as COVID19. Acronyms like tQCMR is difficult to follow in the manuscript, especially since this is a percentage. Please rename the acronyms.	We agree that this is problematic, and we have found it challenging to construct appropriate acronyms for MOAKS and Q-MOAKS. We have made an attempt at a better set of acronyms but kept the basis as “MCM” since this stands for “MOAKS Cartilage Morphometry” (which is the OAI nomenclature) and “QCM” since this stands for “Q-MOAKS Cartilage Morphometry” which is

	in keeping with the editor's suggestion of using an abbreviated version. However, the ThCtAB which stands for "Thickness Cartilage total Area Bone" and the regional cartilage acronyms (eg cMF) have been settled in the literature for many years and we could potentially confuse the readers further if we change those. The "d" in front of MCM is also following this standard cartilage thickness nomenclature meaning "denuded". This cartilage nomenclature is described in: [Eckstein et al (2006). "Proposal for a nomenclature for Magnetic Resonance Imaging based measures of articular cartilage in osteoarthritis." *Osteoarthritis and Cartilage*, 14(10), 974–983.]<br><br>Action: throughout the manuscript we have altered tMCM to ThMCM and altered tQCM to ThQCM to be similar to the ThCtAB nomenclature. We have altered the QCMr ratio nomenclature to QCM% instead. We have also added a diagram to aid interpretation:<br>• See Figure 2.
6. Did any of the cartilage measures scores increase over time?	We thank the editor for this interesting question. Because average changes in thickness of cartilage over a period of one or two years tend to be very small (around 100µm cartilage thickness loss might be expected in 12 months for a progressor group), and the width of a sampling voxel in the MR image is around 300 µm, there is considerable error in the measurement of cartilage thickness either quantitatively or by scoring. Therefore, examining cartilage changes at an individual level is likely within measurement error.<br><br>However, at the group level, we can see in Table 4 that there are a few instances of cartilage increase over time. In particular, over 1 year MOAKS scores decrease in the pLT region and Q-MOAKS scores decrease in the aLT region. Over 2 years, the MOAKS score at the pLT region decreases. There are no instances of the quantitative cartilage thickness measure (ThCtAB) increasing. Given that the actual decreases in these scores are very small, it is likely that these values are reflecting errors in the scores or measures. We have noted this in the DISCUSSION.

	Action: we have added to the DISCUSSION section:<br>• Lines 310-315: …case for Q-MOAKS which showed some variability in this regard. There were a small number of instances of scores indicating cartilage increase: over 1 year MOAKS scores decreased in the pLT region and Q-MOAKS scores decrease in the aLT region. Over 2 years, the MOAKS score at the pLT region decreased. There are no instances of the quantitative cartilage thickness measure (ThCtAB) increasing. Given that the actual decreases in these scores are very small, it is likely that these values are simply reflecting errors in the scores or measures.
7. There is really poor correlation between MOAKS and QMOAKS, with r< 0.6 for all. Why is this? To make QMOAKS supplant MOAKS, you need to show better predictive ability to predict say time to knee replacement or pain or some other important clinical value. As is, the current presentation has no clinical value.	As stated in the Discussion, we believe that this may be because an accurate semi-quantitative evaluation of cartilage status by area of loss or denudation may be difficult to achieve.<br><br>To clarify though, our intention is not to promote Q-MOAKS as an evaluation tool in either the clinic or in clinical trials. It is a simply a quantitative measure we produced for this study only, in order to test the accuracy of and sensitivity of the semi-quantitative MOAKS scoring construct; therefore, its predictive ability is not something we sought to establish.<br><br>We do feel that we have stated this is in the DISCUSSION at lines 321-325: “We constructed a quantitative equivalent to MOAKS (Q-MOAKS) using the same anatomical boundaries as MOAKS to explore how scores based on semi-quantitative and quantitative cartilage measures compare. Q-MOAKS was not designed as a measurement tool itself (and we would not advocate it’s use for that purpose), but to understand the relative responsiveness and associations of semi-quantitative cartilage scores and quantitative cartilage thickness measures.” However, we have added to the Introduction as well, in order to make this clearer.

	Action: We added clarification to the Introduction:<br>• Lines 81-82: …MOAKS. This "Q-MOAKS" construct was designed only to compare quantitative measures to MOAKS and was not intended as a replacement of the scoring system, or as a novel clinical tool. For…
8. Please address/discuss age, sex, and BMI differences between the cases and controls.	We thank the editor for pointing out the fact that we had not drawn attention to the age and BMI difference in the control group.<br><br>Action: we have added some detail in the RESULTS section:<br>• Line182: Our OAI baseline non-OA control group of 549 subject knees had lower BMI but had almost the same proportion of women (52%), however they were younger and had lower BMI which might be expected in a non-OA group.

Reviewer Comments:

Reviewer #1 Comments	Response / Action
This is an interesting manuscript that reports a direct comparison of a quantitative cartilage software measurement method to MOAKS scoring. I have a few general comments. First, it would be helpful for review if the authors numbered the manuscript lines. Parts of the manuscript are written seeming to suggest that Q-MOAKS is the ground truth. e.g. the bottom of Page 8: "poor accuracy", and later "true accuracy" Without an independent measurement like excised cartilage specimens, one cannot definitively claim to know the "true" cartilage volume and/or thickness. Quantitative methods are also subject to errors and biases. In comparing Q-MOAKS to MOAKS, it seems like there is an "apples to oranges" problem as discussed below.	We thank the reviewer for their comments made several changes to the manuscript. We would agree that the Q-MOAKS construct is not a "ground truth" but it is a quantitative version of MOAKS and therefore is a basis for comparison. We have altered the "accuracy" wording to indicate levels of agreement instead.<br><br>Action:<br>• we have added line numbers.<br>• Line 191: However, in general, the boxplots indicate poor ~~accuracy~~ agreement of MOAKS scoring in terms of the…<br>• Line 198-201: In addition, MOAKS appeared to have a bias in that it systematically over-estimates the ~~true~~ area of cartilage affected by quantitative cartilage loss in both tibial and femoral regions.
Page 5. Change "joint space narrowing (JSN)" to "decrease in joint space width (JSW)" so as not to confuse with the OARSI JSN score.	We agree that this would be a useful change and have changed in manuscript.

	Action:<br>• Line 92-93: 0.7mm decrease in joint space ~~narrowing~~ width (JSW~~N~~) in the medial tibiofemoral compartment from…<br>• Line 275: progression, defined as $\geq$0.7mm ~~JSN~~ decrease in JSW in the medial tibiofemoral compartment during…
Page 5. Are some (or all) of the N=549 control knees drawn from the OAI (N=122) Control Cohort?	We thank the reviewer for bringing to our attention that this fact was omitted from the manuscript. We have now added a note in the Methods.<br><br>Action: We have added to the METHODS section:<br>• Lines 96-97: KL grade 0 with no pain (WOMAC pain subscale = 0 or 1), that were not included in the 600 participants of the FNIH Biomarkers study groups.
Page 5. Although they say "read centrally", the authors should clarify that the MOAKS scores were part of the OAI data release. They should also say something about the expertise of the readers.	We thank the reviewer for pointing out this omission. We have made it clear that the MOAKS scores were from the OAI data release and that the readers were both experienced MSK radiologists.<br><br>Action: we have amended the text in the METHODS:<br>Lines 99-102: For semiquantitative scoring in the FNIH study, MR images were ~~read~~ scored unblinded to timepoint using the MOAKS scoring system centrally by two experienced musculoskeletal radiologists with 13- and 15-years' experience of semi-quantitative assessment of knee OA. The MOAKS scores were then made publicly available through the OAI data release. ~~and unblinded to timepoint using the MOAKS scoring criteria.~~
Page 7. Were the Q-MOAKS read blinded or unblinded to time point. If Q-MOAKS is fully automated, then this is a moot point but if there is any human review of the software results, it is relevant.	The Q-MOAKS computation was completely automated, and each image was analyzed independently so there was no concept of blinding. We have clarified this in the METHODS section.<br><br>Action: we have updated the METHODS section:<br>• Line 142: cartilage region, we developed two fully-automated "Q-MOAKS" measures…
Page 7. I am not sure the definition of normative ThCtAB is valid. Was there any stratification for	It could be argued that we should have stratified the normative cartilage thickness by sex and

sex or other factors? It probably is one way to define loss or denudation, but certainly is unlikely to replicate what a MOAKS reader actually does. It is therefore not surprising that MOAKS and Q-MOAKS do not correlate well. The definition of Q-MOAKS is a bit confusing. It appears that Q-MOAKS is ordinal like MOAKS, but "Q-MOAKS percent area" is not. It would be helpful if the authors stated this explicitly.	height, which are known to influence this measure. However, we are skeptical that the radiologist scoring MOAKS takes these factors into account rather than simply looking for apparent thinning. However, we did use point-by-point cartilage thickness measures rather than a global average, which we think is a more appropriate and subtle approach. We then calculated the ratio of cartilage with apparent thinning in each area. So, we do think that this should model quantitatively what MOAKS is trying to achieve with its scoring of percentage of thinned or denuded cartilage. We believe that the correlation is poor because the semi-quantitative evaluation of cartilage status by visual integration of area of loss or denudation may be difficult to achieve.<br><br>In terms of the definition of Q-MOAKS percent area, we have added some detail to make this a clearer.<br><br>Action: we have added some additional wording to define the Q-MOAKS percentage area measure in the METHODS section:<br>• Lines 153-156: …to the cartilage thickness loss score. The Q-MOAKS scores are therefore an ordinal scale, similar to MOAKS, whereas the Q-MOAKS area percentage measures are a continuous measure of the proportion of cartilage loss or denudation over one of the defined cartilage regions. These Q-MOAKS…
Pages 7 and 8: I am not sure whether the SRM is a valid way to compare an ordinal score to a non-ordinal measurement. There are statistical test that look at the ability of a method to correctly order the time points. Such a test may be more appropriate. It would also be good to compare Q-MOAKS with the OAI released quantitative cartilage measurements (Chondrometrics) For such a comparison, the SRM would certainly be valid.	We thank the reviewer for this comment. We agree that while SRMs are often used to assess responsiveness, the interpretation of this measure is less intuitive for non-Gaussian outcomes, making direct comparison between SQ and quantitative measures difficult. This potential violation of normality assumption may warrant the adoption of nonparametric methods rather than their parametric counterparts. Unfortunately, there is very little literature on longitudinal sensitivity to change of SQ scoring systems such as MOAKS and WORMS. To our knowledge the examples that exist use SRM as the sensitivity measure and we aimed to compare

	our study to that previous work and that informed our choice of statistic.<br><br>In terms of the comparison of Q-MOAKS and cartilage thickness measures, there are two previous validation studies that show that the cartilage thickness measure used in this study is comparable to the Chondrometrics measures: these are cited in references [5] and [6]. However, the Chondrometrics measures use cartilage ROIs which have become standard but are different from the MOAKS regions. Therefore, Chondrometrics measures are not directly comparable to Q-MOAKS measures or scores. In contrast, in the present study, we were able to adapt our automated cartilage measures to report the same area regions as those use for MOAKS.<br><br>We have added a brief description of the validation of our automated method with Chondrometrics manual segmentation with the FNIH biomarkers set of 600 subjects in the Methods section.<br><br>Action: We have added to the METHODS section:<br>• Lines 124-126: Validation of this method showing comparable thickness measurements to manual segmentation using image data from all 600 subjects in the OA Biomarkers consortium FNIH study in the FNIH has been previously reported [6]. The mean AAM shape was annotated to produce bone surface regions…

Reviewer #2 Comments	Response / Action
METHODS<br>All the acronyms and measurements are difficult to follow. Figure with example of measurements will be useful.	We agree that this is problematic, and we have found it challenging to construct appropriate acronyms for MOAKS and Q-MOAKS. We have made an attempt at a better set of acronyms but kept the basis as “MCM” since this stands for “MOAKS Cartilage Morphometry” (which is the OAI nomenclature) and “QCM” since this stands for “Q-MOAKS Cartilage Morphometry”. However, the ThCtAB which stands for “Thickness Cartilage total Area Bone” and the regional cartilage

	acronyms (eg cMF) have been settled in the literature for many years and we could potentially confuse the readers further if we change those. The “d” in front of MCM is also following this standard cartilage thickness nomenclature meaning “denuded”. This cartilage nomenclature is described in: [Eckstein et al (2006). “Proposal for a nomenclature for Magnetic Resonance Imaging based measures of articular cartilage in osteoarthritis.” *Osteoarthritis and Cartilage*, 14(10), 974–983.]. WE agree that a figure would help and have added one to the METHODS section.<br><br>Action: throughout the manuscript we have altered tMCM to ThMCM and altered tQCM to ThQCM to be similar to the ThCtAB nomenclature. We have altered the QCMr ratio nomenclature to QCM% instead. We have also added a diagram to aid interpretation:<br>• See Figure 2.
RESULTS<br>Cross-sectional findings<br>Age and BMI of the FNIH groups and non-OA group are rather different. In particular, age is important risk factor of OA and there is no overlap. Are you using the non-OA group as "normal control" to calculate degree of cartilage thinning? Please be more explicit. Regardless, there is no "normal" cartilage thickness for each age group established. The lack of "normal" should be noted in the discussion.	We thank the reviewer for raising this issue, and we agree that it could be argued that we should have stratified the normative cartilage thickness by age (or indeed sex and height), which are known to influence this measure. Age of course is a risk factor for OA, and so cartilage may be somewhat thinner in an older population. Our intention was to find a set of control knees that had no radiographic OA and no pain to establish a comparator mean thickness value for cartilage thickness loss, regardless of age. Within the OAI baseline set, it is apparent that this produces a sample that is younger than our JSW progressor set.<br><br>Therefore, we are indeed using the non-OA group as "normal control" to calculate degree of cartilage thinning. Obviously, we cannot stratify by age because there is no overlap in the two groups, however, our intention in using this group was to define thresholds for probable thinning and denudation and we believe it is valid for that. We have made this clearer in the METHODS and added some narrative about the concept of “normal” in the DISCUSSION.

	Action: We have added detail on the use of the non-OA group to define thresholds for thinning and denudation in the METHODS and added some narrative about the concept of "normal" in the DISCUSSION:<br>• Lines 142-143: …cartilage region, we developed two "Q-MOAKS" measures. We used the non-OA control group to define normative cartilage thickness values for comparison. First, normative cartilage thickness values were…<br>• Lines 361-367: There are a several limitations of this work. The normative cartilage thickness values for the determination of thinning and denudation we derived from the non-OA group was not stratified by sex or height, both of which may alter expected thickness. We might also have stratified by age, but this was not possible as there was little or no overlap between the non-OA and FNIH biomarker groups. These stratifications might have made the Q-MOAKS scores more accurate. However, our intention was not to define a set of "normal" cartilage thickness measures, but to compute simple thresholds to determine probable thinning and denudation. Future work might improve upon this. The study…
DISUCSSION<br>Is there a reason there were no MOAKS grade 3 denuded knees? It is a bit difficult to think for participants aged up to 69 there were no one with grade 3. Were they actively excluded? If so, why?	We agree with the reviewer, and it is surprising given that we chose the two groups of subjects from the FNIH biomarkers initiative that had confirmed JSW loss beyond measurement error (0.7mm). However, the FNIH biomarkers initiative excluded KL grade 4 subjects since these are categorized as "end-stage disease", and these would have had a higher proportion of large denuded cartilage areas in them. The MOAKS data was a public release form the Osteoarthritis Initiative and the MR images were read by two very experienced MSK radiologists familiar with SQ scoring and so we have confidence in this data. We did not exclude any of the data from these two groups. We have added the KL grade 4 exclusion criteria in the METHODS section.

	Action: we have added the KL grade 4 exclusion criteria in the METHODS section:<br>• Line 88-89: Four knee outcome groups comprising 600 participants with knee Kellgren-Lawrence (KL) grade 1-3 and with combinations of radiographic and/or pain…
